# Neuropathic Symptoms and Frequency of Chronic Pain in an International Online Sample of Individuals with Sub-Acute and Chronic Stroke

**DOI:** 10.3390/healthcare13050455

**Published:** 2025-02-20

**Authors:** Brendon S. Haslam, David S. Butler, Anthony S. Kim, Leeanne M. Carey

**Affiliations:** 1 Occupational Therapy, School of Allied Health, Human Services and Sport, La Trobe University, Melbourne, VIC 3086, Australia; l.carey@latrobe.edu.au; 2 Neurorehabilitation and Recovery, The Florey, University of Melbourne, Melbourne, VIC 3001, Australia; 3 IMPACT in Health, University of South Australia, Kaurna Country, Adelaide, SA 5001, Australia; 4 Neuro Orthopaedic Institute, Adelaide, SA 5001, Australia; 5 Weill Institute of Neurosciences, Department of Neurology, University of California, San Francisco, CA 94143, USA

**Keywords:** chronic pain, neuropathic pain, pain, stroke

## Abstract

**Background/Objectives**: Chronic pain is common following a stroke and is associated with increased disability. Yet, little is known about the chronic pain experience in the stroke population. This study aimed to identify and explore the features and neuropathic symptoms of chronic pain in individuals with longstanding stroke. **Methods**: This observational study utilized an online survey that was developed for individuals who have had a stroke (>3 months). Data sought included participant demographics, medical history, and details of the stroke(s). Participants who reported experiencing chronic pain completed the Numerical Rating Scale for Pain, the Neuropathic Pain Symptom Inventory, and body maps to indicate region(s) of pain. **Results**: A total of 533 individuals with longstanding stroke participated. Chronic pain was reported as being experienced by 60% of participants and was more frequently experienced by individuals who reported being female (*p* = 0.002). Moderate or severe pain intensity was commonly reported (mean = 5.98, *SD* = 1.89). Individuals with chronic pain post-stroke reported a range of neuropathic symptoms rather than a common pain experience, with combinations of spontaneous, paroxysmal, and evoked pains in addition to pain associated with paraesthesia/dysaesthesia. Pain involving the upper limb was the most common region (shoulder 39%, hand and forearm 38%), followed by the lower limb (foot 30%, leg 29%). Having multiple strokes was associated with a higher frequency of chronic pain (*p* = 0.01), as was peripheral vascular disease (*p* < 0.001) and lipid disorders (*p* = 0.001). **Conclusions**: These findings highlight the varied nature of chronic pain experienced by individuals following a stroke, while also detailing stroke and medical history associated with chronic pain. It builds on existing knowledge of chronic pain post-stroke and provides new insight into the neuropathic symptoms experienced. This knowledge has the potential to assist in the development of tailored interventions based on addressing pain symptomatology and health literacy.

## 1. Introduction

Stroke is a leading cause of adult disability and the second most common cause of death [1,2]. The burden of disability due to stroke has recently increased due to a substantial decrease in stroke mortality rates [3], resulting in increased numbers of stroke survivors living in the community.

In addition to the disability experienced by stroke survivors due to impairments that are a direct result of the stroke, such as difficulties in motor function and mobility, speech disorders, and sensory impairments [3], many individuals (40–65%) develop chronic pain in the weeks to months following their stroke and continue to experience it [4,5]; where chronic pain is defined as “pain that persists or recurs for longer than 3 months” [6]. This is more than double the frequency of chronic pain reported in the general population [7,8]. Individuals with chronic pain post-stroke commonly experience higher rates of depression, fatigue, and anxiety in addition to difficulties in cognitive function and physical activity than those without chronic pain [9,10], and treatment of chronic pain is identified as an unmet need by survivors of stroke [11].

Studies reporting post-stroke pain frequently refer to pain by the allocation of a descriptive diagnosis (e.g., central post-stroke pain, post-stroke shoulder pain) [12,13] or type (e.g., nociceptive/musculoskeletal, neuropathic) [14,15]. These observational studies are often undertaken when stroke survivors are continuing to access post-stroke hospital-based services such as rehabilitation [16], or at designated time points post-stroke as part of a stroke registry follow-up [5,14,17].

As in other chronic pain conditions, it is likely that in post-stroke chronic pain, there will be multiple contributing factors to the experience of pain. These may be a direct result of the stroke itself, including, but not limited to, injury to the nervous system and altered sensorimotor function [18]. However, these factors may be considered in addition to predisposing factors such as depression [19] or factors associated with likely adaptive processes that may occur over time following stroke, consistent with the development of altered body postures or spasticity [18]. Adaptive neural processes such as changes in functional cortical organization [20] and central sensitization [21] are known to occur in non-stroke chronic pain conditions, and these are also thought to occur post-stroke [22]. Chronic pain post-stroke can therefore be due to various combinations of types of pain (e.g., nociceptive, neuropathic, and nociplastic) as defined by the International Association for the Study of Pain [23].

Given the likelihood of potential changing contributions and mixed pain experienced by stroke survivors, it is surprising that there is little known about the stroke survivor’s ongoing experience of pain in the community. The objectives of this study were to identify the frequency of chronic pain reported by stroke survivors in the community beyond what is currently known for populations actively engaged in stroke-related health services, identify the features of chronic pain experienced by individuals post-stroke (e.g., pain symptoms often reported by individuals with neuropathic contributions towards their pain, body regions, and severity), and explore potential associations of chronic pain relevant to the nature of stroke, sex, and medical history.

It is proposed that the identification of neuropathic symptomatic features commonly associated with chronic pain post-stroke will lead to the overall aim of a greater understanding of chronic pain in stroke and contribute to the development of tailored treatment strategies for chronic pain post-stroke, targeting different pain symptoms that are experienced. Improved understanding of associations existing between medical conditions and chronic pain post-stroke may also help to identify at-risk individuals post-stroke and enable the implementation of strategies aimed at preventing the development of chronic pain in future populations of stroke survivors.

## 2. Materials and Methods

This observational study was part of a larger research program, “RECOGNISE”. “RECOGNISE” was conducted via an online survey. It contained multiple questionnaires in English and interactive tasks related to body schema designed for individuals who have had a stroke. This study was approved by the Human Research Ethics Committee of the University of Melbourne, Australia (Ethics ID 1340670), the Human Ethics Committee of La Trobe University, Melbourne, Australia, and the Institutional Review Board of the University of California, San Francisco, United States of America. Funding for software development and website hosting was provided by the Neuro Orthopaedic Institute, Australasia. Participation was possible using either a tablet or laptop/desktop computer at a time and location of convenience for the participant. A protocol outlining the analysis plan was developed and lodged with Open Science Framework [24] prior to the analysis of the full data set. Results from “RECOGNISE” have previously been published related to pain beliefs and perceptions [25], utilization of pain treatments [26], body schema [27], and perception [28], and the relationship between somatosensory impairment and chronic pain in longstanding survivors of stroke [29]. The purpose of using “RECOGNISE” for this study was: to identify the frequency of individuals who experience chronic pain in a population of online users who have had a stroke; to identify the neuropathic symptoms of pain reported; to identify the severity of pain reported; and to identify any potential associations of chronic pain relevant to the individuals’ stroke and medical history. An option was provided for the survey to be completed by another party. The anonymous survey sought demographic details (year of birth, sex, and country of residence) and self-reported basic medical conditions that they had been diagnosed with, including details of their stroke(s). Participants were asked if they had experienced pain for the past three months or more, and if so, they were asked to complete the Numerical Rating Scale for Pain (NRS) and the Neuropathic Pain Symptom Inventory (NPSI) [30]. Pilot trials were performed by individuals with and without stroke; participation took approximately 14–20 min. From November 2016, participants were also asked to complete body charts identifying body regions associated with chronic pain if experienced. Data were collected between October 2015 and October 2018.

### 2.1. Subjects

Participants were eligible to be included in the study if they reported having been diagnosed with one or more strokes at least three months previously, were eighteen years of age or older, and were able to provide consent. The nominated time-point of three months was to reflect the nature of pain post-stroke being developed in the weeks to months following the stroke. Informed consent to participate in the study was obtained from all participants. To complete the survey, eligible participants were required to have adequate online technology access and written English skills. Potential participants were excluded if they were not able to access the study due to technology limitations, were younger than eighteen years, had not been diagnosed with a stroke more than three months prior, and were either unable to or chose not to provide consent.

### 2.2. Recruitment Procedure

Potential participants were made aware of the study through an established research register for stroke survivors who had indicated a willingness to participate in research activities, in addition to the study being listed on websites and in newsletters of agreeing stroke-related associations.

### 2.3. Measures

Numerical Rating Scale for Pain (NRS): Participants were asked to score their average severity of pain by indicating a number from 0 to 10, with 0 labeled as “no pain” and 10 as “worst pain imaginable”. The NRS has been shown to be a valid, reliable, and sensitive measure for pain [31] and has previously been used in large online studies [32]. The clinical utility of the NRS has been demonstrated in stroke survivors who are able to collaborate and communicate, a requirement of self-reported scales. It was preferred to the Faces Pain Scale and Visual Analogue Scale by people who have had a stroke [33], while still positively correlating with both.

Neuropathic Pain Symptom Inventory (NPSI): The NPSI [30] was used for all participants who reported experiencing chronic pain, to identify the prevalence and severity of the multiple pain symptoms commonly described by individuals who have had a stroke who are considered neuropathic in nature. The NPSI is a measure designed specifically for conditions where neuropathic characteristics of pain are likely, presenting different descriptors of pain symptoms and asking participants to rate each pain symptom on a 0 to 10 numerical rating scale. It has been validated for use in individuals who have had a stroke, a condition where neuropathic characteristics are likely to be experienced [30].

Body Maps: Participants from November 2016 were asked to indicate any regions where pain was experienced using radar buttons on body maps.

### 2.4. Data Analysis

Participants were divided into two groups: those with chronic pain and those without. Means were calculated for age and for duration since the first stroke, and differences compared between groups (Student *t*-test). Sex, prior handedness, presence of chronic pain prior to stroke, and experience of single or multiple strokes were summed according to frequency and compared (Chi-square test). Means and standard deviations were calculated for the intensity scores of the chronic pain group for the NRS and the NPSI. The intensity of pain, as indicated by the NRS score, was also categorized as mild, moderate, or severe pain, as described by Boonstra et al. [31]. Statistical analyses were performed using the IBM SPSS Statistics (https://www.ibm.com/spss), with significance levels set at *p* < 0.05. Between-group differences for diagnoses of other medical conditions were deemed significant at *p* < 0.007 following correction for multiple comparisons. This manuscript conforms to the STROBE (Strengthening the Reporting of Observational Studies in Epidemiology) guidelines [34].

## 3. Results

A total of 533 individuals who have had a stroke participated in the study, with 60% (*n* = 319) reporting that they experienced chronic pain, compared to 40% (*n* = 214) who reported that they did not. The majority of participants were from the United States of America (315), followed by Australia (123). All surveys were completed by the participating individual who had a stroke, despite the option for it to be completed by other parties. The mean age and chronicity of stroke are indicated in Table 1, along with the distribution of handedness and identified gender. Twenty percent of those with chronic pain indicated that they had experienced chronic pain prior to their stroke, compared to nine percent of those without (*p* = 0.002). Individuals who reported being female reported a higher frequency (57%) of experiencing chronic pain than those who did not (43%).

### 3.1. Pain Symptoms

The mean reported pain intensity for individuals with chronic pain was 5.98 out of 10 (SD = 1.89). Fifty-nine percent reported moderate or severe pain. Participants with chronic pain experienced a mix of pain symptoms, with all participants reporting that they experienced at least one of the neuropathic pain symptoms described in the NPSI. Symptoms associated with paraesthesia/dysaesthesia (pins and needles 65%, tingling 70%) were the most common, and cold-evoked pain the least (47%). Reported intensity means for each NPSI item were similar, ranging between 5.18 and 6.09 out of 10. Prevalence and mean reported intensity of NPSI items are reported in Table 2.

Higher pain intensity was reported by female participants with chronic pain; however, no differences were detected in the frequency of neuropathic symptoms between sexes (see Table 3).

While having multiple strokes was found to be more likely associated with increased frequency of chronic pain, no differences were observed between single and multiple strokes for pain intensity or frequency of neuropathic symptoms (see Table 4).

Participants from November 2016 (*n* = 380) were asked to indicate any body regions where they experienced chronic pain symptoms. Pain experienced in the upper limb (shoulder and hand/forearm) was most common, followed by lower limb (leg and foot), back, and neck pain. The frequency of pain region as reported by participants post-November 2016 is indicated in Table 5.

### 3.2. Stroke and Medical History Factors

Having multiple strokes (2 or more) was associated with an increase in the presence of chronic pain (*p* = 0.01). The reported frequency of the number of strokes is presented in Table 6. No difference between the two groups was observed for frequency of reported stroke type or lesion side; however, many participants did not respond to these questions or indicated that they did not know the stroke type or side of the lesion.

Differences in reported medical history were found between groups. Diagnosis of lipid disorders (*p* = 0.001) and peripheral vascular disease (*p* < 0.001) were higher in those with chronic pain. A diagnosis of complex regional pain syndrome (CRPS) was given for ten percent of those with chronic pain, while three individuals (one percent) of those who were no longer experiencing chronic pain reported that they had previously been diagnosed with CRPS. No differences were detected in the other suggested medical conditions, as reported in Table 6.

## 4. Discussion

Our findings expand on previous studies of post-stroke chronic pain by exploring neuropathic pain symptomatology and potential contributing factors in individuals who have had a stroke across their lifespans. Our sample population was younger (mean age 58.9 years) than reported in other studies [4,5,17] possibly due to the online nature of the survey. Experiencing a stroke at a younger age has been reported to have an increased likelihood of developing chronic pain [5,35] and may explain why the reported frequency of chronic pain in our sample was in the higher range compared to other studies. Twenty percent of those experiencing chronic pain reported chronic pain prior to their stroke, consistent with findings of the general population in the two countries of highest recruitment (i.e., the United States of America and Australia) for this study [7,8], suggesting no predisposition to chronic pain in our sample population, and supporting previous findings of the development of novel pain post-stroke [36]. Our finding of the 9% of participants who had a reversal of pain following a stroke (i.e., no longer experienced chronic pain) warrants further exploration. Consistent with findings in other chronic pain populations [7,8,37], females in this study population reported higher rates of experiencing chronic pain and reported it as being higher in intensity [38]. Some of the potential interactions of multiple contributing factors such as genetic expression, hormones, and immune response for this observed difference in sex in chronic pain states have been identified and continue to be studied in greater depth [39] in chronic pain states, including those with neuropathic contributions [40].

Shoulder pain was the most common body region (39%) indicated by participants with chronic pain. This is consistent with previous studies that have described hemiplegic shoulder pain as one of the four most common medical complications following a stroke [16]. The development of shoulder pain following a stroke occurs over time, with only 10% experiencing shoulder pain in the acute phase [41]. Considered together, these findings support the development of pain following a stroke, such as shoulder pain, involving contributions from neural adaptive processes over time, such as central sensitization [22].

Neuropathic pain is to be considered as being more of a clinical description than a diagnosis, being defined by the International Association for the Study of Pain as “pain caused by a lesion or disease of the somatosensory nervous system” [23]. While there are established assessments to determine the likely presence of neuropathic pain characteristics, such as the Douleur Neuorpathique 4 (DN4) [42] and the Leeds Assessment of Neuropathic Symptoms and Signs (LANSS) [43], they do not have the ability to identify the broad range of symptoms often described in neuropathic pain conditions or their severity. It is recommended that for pain conditions with likely neuropathic characteristics that, a combination of measures is used to identify potential mechanisms that may be associated with neuropathic-type symptoms [44]. This should include a pain intensity measure such as the NRS or Visual Analogue Scale and a pain quality measure such as the NPSI or Neuropathic Pain Scale [44]. In wanting to explore neuropathic pain symptomatology and severity experienced by stroke survivors and be consistent with current recommendations, the NPSI was chosen for this study, and all stroke survivors who reported chronic pain were asked to complete it. In doing this, it was possible to capture the presence of neuropathic symptoms in more descriptive detail (e.g., spontaneous, paroxysmal, evoked, and paraesthesia/dysaesthesia), which may help ultimately in the identification of differing mechanisms that contribute to the chronic pain experience post-stroke and provide guidance towards the development of targeted therapies (e.g., different therapeutic approaches may be appropriate for evoked pain compared to non-evoked spontaneous pain or pain associated with numbness).

Findings from our study highlight the varied presentations and experiences of individuals with chronic pain, which frequently included a mix of spontaneous, paroxysmal, and evoked symptoms in addition to painful symptoms associated with paraesthesia/dysaesthesia. Pain symptoms associated with paraesthesia/dysaesthesia were experienced most in our population, regardless of potential pain type diagnosis. This is consistent with a previous study utilizing the NPSI in central post-stroke pain [45], while a study with a smaller sample size (*n* = 43) investigating characteristics of pain and potential aggravating factors of individuals at two years post-stroke also found that pain was commonly increased by evoking factors such as cold or being touched [46]. Further analysis is required to see if there are profiles of pain symptoms experienced by individuals with chronic strokes, as has been found in other neurological conditions such as painful diabetic neuropathy [47].

No difference in self-reported type of stroke (ischemic or hemorrhagic) between groups was found. It has been reported that ischemic stroke was more frequently associated with central post-stroke pain than hemorrhagic stroke [12]; however, it is important to highlight that central post-stroke pain is considered to be present in only 7–8% of individuals who have had a stroke [12,48]. An important aim of our study was to be inclusive of all individuals who have had a stroke and experienced chronic pain, recognizing the potential multiple contributions towards the pain experienced in a chronic stroke population.

Fourteen percent of participants indicated ‘unknown’ or chose not to respond to questions related to the nature of their stroke, indicating a possible lack of information or understanding about their stroke and medical conditions. This identifies a potential role for improved education of stroke survivors in understanding the nature of their stroke and other medical conditions, improving their ability to independently manage their health (i.e., health literacy). Education aimed at improving health literacy has been suggested as a potential strategy in the treatment of chronic pain in strokes [25] and is consistent with educational strategies in the evidence-based treatment of other chronic pain conditions such as whiplash [49], fibromyalgia [50], and chronic musculoskeletal pain [51].

### 4.1. Limitations

To participate, individuals were required to have internet access, adequate technology skills, and an understanding of the English language to enable them to complete the survey independently. Individuals with aphasia were assumed unable to participate due to the nature of the online study, despite evidence that individuals with aphasia also experience high rates of pain [52,53]. While the survey included provisions to be completed by other parties, this option was not utilized by participants in the study. This study was promoted through multiple avenues over a period of three years. It will have been inaccessible to many, and, therefore, the sample reported may not be a true representation of the chronic stroke population. In utilizing the recruitment strategy outlined, we were unable to determine the response rate for this study but were able to determine a completion/non-completion rate. Our non-completion rate of four percent compares favorably to expected rates for non-incentive online studies, which are often associated with incomplete data [54]. Being survey-based, it relied on self-reporting and, therefore, did not have the capacity to include any diagnostic investigations to determine the type or side of stroke. It is possible that some inaccuracies in reporting or a lack of reporting (as discussed earlier) occurred due to a lack of knowledge or understanding. We also found an association in our chronic pain post-stroke group with self-reported peripheral vascular disease. Peripheral vascular disease is also associated with an increased prevalence of chronic pain [55], and our study was not able to distinguish between a stroke and peripheral vascular disease as likely causes of, or contributions to, the pain experience. Further, we are not able to identify if the self-reported diagnoses were present at the time or associated with an earlier time and no longer present when the individual participated in the study. The survey questions were also not able to identify the duration that the individuals who had a stroke had experienced pain, as it only asked if they had experienced pain for a period of three or more months.

### 4.2. Conclusions

Our findings highlight the common problem of chronic pain post-stroke as an international issue, with individuals commonly reporting moderate or severe pain. Despite this high prevalence and severity, understanding of the pathophysiology and, thus, development of effective treatments remains largely unaddressed, despite headway being made in other chronic pain populations such as CRPS.

We found that while upper limb pain was most common, the prevalence of lower limb, back, and neck pain was also high; thus, post-stroke pain and potential treatments should be considered in the context of the whole body rather than the current focus on regions and structures such as the glenohumeral joint of the shoulder in clinical guidelines [56]. This finding also supports the proposal that the development of pain over time may occur through processes such as central sensitization or upregulation [22,57] or the development of muscle spasticity [18] rather than purely in response to multiple injuries or joint degeneration.

### 4.3. Implications

With greater knowledge and understanding about the nature of the chronic pain experienced in longstanding strokes, and the variability of the symptoms experienced, much of the phenomena of chronic pain post-stroke can be de-threatened. This information can be part of an intervention program that includes an educational strategy for stroke survivors (i.e., the individual who has had a stroke will not associate each experience of pain with new or recurrent injury). Educational approaches have been shown to have positive effects in the treatment of chronic pain in other conditions [58,59] and can be included as part of a combined therapeutic approach that aims to improve movement and function [60]. The individual with pain could be provided with education related to their pain supported by the knowledge that pain post-stroke is often developed over time as a result of adaptive processes rather than supporting the fixed belief that their stroke has directly caused their pain [25]. In addition, this knowledge of adaptive processes may lead to being more open to proposed strategies and treatment options while describing how they are aiming to help the individual in managing their pain and increasing function. Knowledge of the different types of symptoms experienced (e.g., spontaneous, evoked) may ultimately assist in directing different therapeutic approaches (e.g., sensory-based rehabilitation aiming to improve somatosensory discrimination of different stimuli) based on these symptoms.

## Figures and Tables

**Table 1:** Frequency and characteristics of stroke sample with and without self-reported chronic pain.

Participants (*n* = 533)	Stroke Pain	Stroke No Pain	*p* Value
Participant group (%)	319 (60%)	214 (40%)	*p* < 0.001 ^a^
Age, years (SD)	58.9 (12.1)	56.4 (13.4)	*p* = 0.028 ^b^
Sex, Female (%)	180/319 (57%)	91/214 (43%)	*p* = 0.002 ^a^
Handedness, Right (%)	274/319 (86%)	172/214 (81%)	*p* = 0.09 ^a^
Duration Since First Stroke, years (SD)	6.1 (6.8)	7.1 (7.0)	*p* = 0.13 ^b^
Prior Chronic Pain (%)	63/319 (20%)	21/214 (9%)	*p* = 0.002 ^a^

^a^ Chi-Square Test; ^b^ Student *t*-test.

**Table 2:** Features of the pain experience.

Pain Feature	Frequency (%)	Intensity Mean (SD)
Pain Intensity: NRS		5.98 (1.89)
Mild Pain (≤5)	132/319 (41%)	
Moderate Pain (6–7)	120/319 (38%)	
Severe Pain (≥8)	67/319 (21%)	
Neuropathic Pain Symptom		
Superficial Spontaneous		
*Burning*	198/319 (62%)	5.56 (3.28)
Deep Spontaneous		
Squeezing	152/319 (48%)	5.29 (3.19)
Pressure	207/319 (65%)	5.84 (3.44)
Paroxysmal		
Electric Shocks	151/319 (47%)	5.38 (3.18)
Stabbing	177/319 (55%)	5.86 (3.50)
Evoked		
Brushing	161/319 (50%)	5.18 (3.20)
Pressure	202/319 (63%)	5.77 (3.45)
Cold	151/319 (47%)	5.68 (3.40)
Paraesthesia/Dysaesthesia		
Pins and Needles	206/319 (65%)	5.87 (3.52)
Tingling	222/319 (70%)	6.09 (3.57)

**Table 3:** Pain symptoms by sex.

Pain Symptom	Female	Male	*p* Value
Pain Intensity NRS, Mean (SD)	6.22 (1.95)	5.72 (1.85)	*p* < 0.026 ^b^
Frequency of neuropathic symptoms			
• Spontaneous	78%	77%	*p* = 0.852 ^a^
• Paroxysmal	63%	63%	*p* = 0.980 ^a^
• Evoked	75%	66%	*p* = 0.098 ^a^
• Paraesthesia/Dysaesthesia	71%	74%	*p* = 0.669 ^a^

NRS = Numerical Rating Scale for Pain; ^a^ Chi-Square test; ^b^ Student *t*-test.

**Table 4:** Pain symptoms by frequency of stroke.

Pain Symptom	Single	Multiple	*p* Value
Pain Intensity NRS, Mean (SD)	5.95 (1.89)	6.25 (1.92)	*p* = 0.257 ^b^
Frequency of neuropathic symptoms			
• Spontaneous	(82%)	(78%)	*p* = 0.859 ^a^
• Paroxysmal	(65%)	(66%)	*p* = 0.820 ^a^
• Evoked	72%	71%	*p* = 0.785 ^b^
• Paraesthesia/Dysaesthesia	(74%)	(75%)	*p* = 0.797 ^a^

NRS = Numerical Rating Scale for Pain; ^a^ Chi-Square test; ^b^ Student *t*-test.

**Table 5:** Body region of reported chronic pain.

Body Region	Frequency (*n* = 380)	Percentage
Shoulder	149	39%
Hand and Forearm	143	38%
Foot	115	30%
Leg	109	29%
Back	93	24%
Neck	66	17%
Face	39	10%
Headache	31	8%
Chest and Abdomen	24	6%

**Table 6:** Stroke characteristics and related medical conditions reported.

	Stroke Pain *n* = 319	Stroke No Pain *n* = 214	*p* Value
Number of Strokes			
• 1	251 (79%)	186 (87%)	*p* = 0.010 ^a^
• 2	41 (13%)	14 (7%)	
• 3	15 (5%)	6 (3%)	
• 4	12 (4%)	8 (4%)	
Type of Stroke			
• Infarct	177 (55%)	94 (44%)	*p* = 0.090 ^a^
• Haemorrhage	83 (26%)	54 (25%)	*p* = 0.839 ^a^
• Both	15 (5%)	14 (7%)	*p* = 0.359 ^a^
• Unknown/Did Not Respond	44 (14%)	52 (24%)	
Side of Lesion			
• Right	151 (47%)	91 (43%)	
• Left	112 (35%)	69 (32%)	
• Both	24 (8%)	9 (4%)	
• Unknown/Did Not Respond	32 (10%)	45 (21%)	
Diagnosed Medical Conditions			
• Hypertension	117 37%)	69 (32%)	*p* = 0.292 ^a^
• Diabetes	55 (17%)	21 (10%)	*p* = 0.016 ^a^
• Peripheral Vascular Disease	51 (16%)	14 (7%)	*p* < 0.001 ^a^
• Atrial Fibrillation	36 (11%)	18 (8%)	*p* = 0.170 ^a^
• Complex Regional Pain Syndrome	33 (10%)	3 (1%)	*p* < 0.001 ^a^
• Lipid Disorders	32 (10%)	6 (3%)	*p* = 0.001 ^a^
• Ischemic Heart Disease	25 (8%)	6 (3%)	*p* = 0.015 ^a^

^a^ Chi-Square test.

## Data Availability

The data presented in this study are available on request from the corresponding author. The data are not publicly available due to planned further analyses.
